# *Theileria annulata* SVSP455 interacts with host HSP60

**DOI:** 10.1186/s13071-022-05427-z

**Published:** 2022-08-30

**Authors:** Zhi Li, Junlong Liu, Shuaiyang Zhao, Quanying Ma, Zhihong Guo, Aihong Liu, Youquan Li, Guiquan Guan, Jianxun Luo, Hong Yin

**Affiliations:** 1 State Key Laboratory of Veterinary Etiological Biology, Key Laboratory of Veterinary Parasitology of Gansu Province, Lanzhou Veterinary Research Institute–Chinese Academy of Agricultural Science, Xujiaping 1, Lanzhou, Gansu 730046 People’s Republic of China; 2 Qinghai Academy of Animal Sciences and Veterinary Medicine, Qinghai University, Xining, Qinghai 810016 People’s Republic of China; 3 Jiangsu Co-Innovation Center for Prevention and Control of Important Animal Infectious Diseases and Zoonoses, Yangzhou, 225009 People’s Republic of China

**Keywords:** *Theileria annulata*, SVSP455, Interaction, Transformation, Host cell apoptosis

## Abstract

**Background:**

*Theileria annulata*, a transforming parasite, invades bovine B cells, dendritic cells and macrophages, promoting the uncontrolled proliferation of these cells. This protozoan evolved intricate strategies to subvert host cell signaling pathways related to antiapoptotic signaling to enable survival and proliferation within the host cells. However, the molecular mechanisms of the cell transformation induced by *T. annulata* remain largely unclear. Although some studies have predicted that the subtelomere-encoded variable secreted protein (SVSP) family plays roles in host-parasite interactions, the evidence for this is limited.

**Methods:**

In the present study, the SVSP455 (*TA05545*) gene, a member of the SVSP gene family, was used as the target molecule. The expression pattern of SVSP455 in different life-cycle stages of *T. annulata* infection was explored using a quantitative real-time PCR assay, and the subcellular distribution of SVSP455 was observed using confocal microscopy. The host cell proteins interacting with SVSP455 were screened using the Y2H system, and their interactions were verified in vivo and in vitro using both bimolecular fluorescence complementation and confocal microscopy, and co-immunoprecipitation assays. The role played by SVSP455 in cell transformation was further explored by using overexpression, RNA interference and drug treatment experiments.

**Results:**

The highest level of the SVSP455 transcript was detected in the schizont stage of *T. annulata*, and the protein was located both on the surface of schizonts and in the host cell cytoplasm. In addition, the interaction between SVSP455 and heat shock protein 60 was shown in vitro, and their link may regulate host cell apoptosis in *T. annulata*-infected cells.

**Conclusion:**

Our findings are the first to reveal that *T. annulata*-secreted SVSP455 molecule directly interacts with both exogenous and endogenous bovine HSP60 protein, and that the interaction of SVSP455-HSP60 may manipulate the host cell apoptosis signaling pathway. These results provide insights into cancer-like phenotypes underlying *Theilera* transformation and therapeutics for protection against other pathogens.

**Graphical Abstract:**

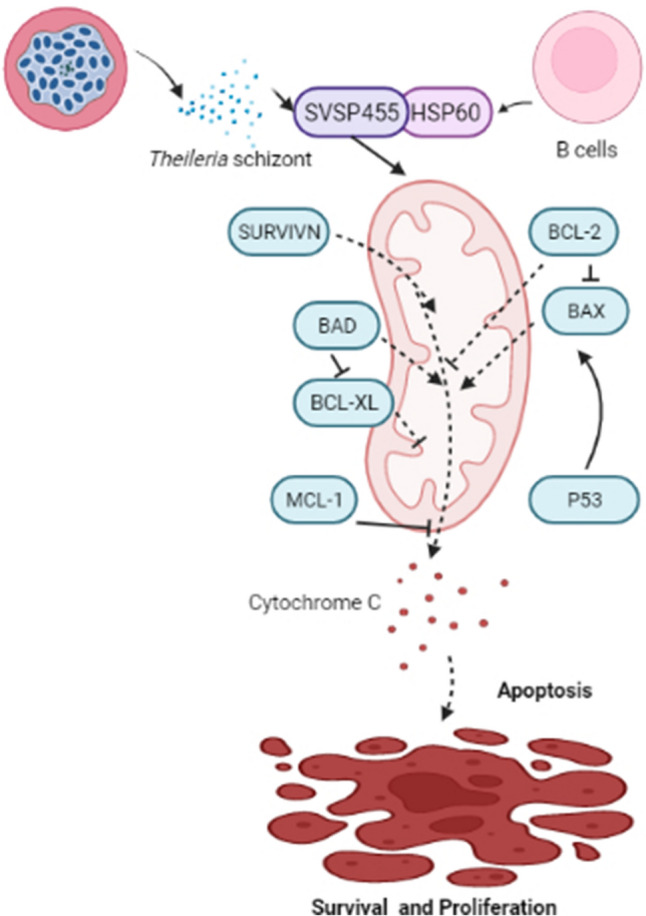

**Supplementary Information:**

The online version contains supplementary material available at 10.1186/s13071-022-05427-z.

## Background

*Theileria annulata*, the pathogen causing bovine tropical theileriosis, leads to acute lymphoproliferative disease in bovines that has a number of clinical features in common with human leukemias [1]. The disease poses a major threat to cattle in subtropical and tropical countries, including Africa, Europe, the Mediterranean basin, the Middle East and Asia [2–8]. Tropical theileriosis also causes substantial economic loss in the livestock industry because it results in mortality in susceptible animals [9, 10] and high costs for the acquisition of anti-tick drugs and treatments [11]. Unlike other apicomplexan parasites, *T. annulata* and *Theileria parva* infect bovine leukocytes and induce the transformation of host cells [12, 13], but the cell tropism of each of these two parasites is distinct. Specifically, *T. parva* invades host T and B lymphocytes whereas *T. annulata* transforms bovine dendritic cells, B cells and macrophages [14, 15]. Although both of these two transforming species infect host B cells, researchers have not yet elucidated whether the transformation mechanism is the same in both species. More importantly, the phenotypes of *Theileria*-transformed cells share some properties with cancer cell hallmarks, including uncontrolled proliferation, increased invasiveness and metastasis and inhibition of cell death [1, 16]. Of particular interest, *T. annulata-*transformed cells lose their cancer-like phenotypes and rein apoptosis sensitivity when treated with the antiparasitic drug buparvaquone (Bup) in vitro [1, 11].

In *Theileria*-induced transformation, several host cell signaling pathways are activated that are involved in controlling parasite proliferation and survival, including the NF-kB, c-Jun N-terminal kinase (JNK) and c-Myc signaling pathways [1, 17–19], but the exact mechanism remains unclear. Decades of research has resulted in the identification of many parasite proteins that contribute to transformation, including TaPIN1 [20, 21], TaSP [22, 23] and Ta-p104 [24]. However, the molecules that play a key role in transformation remain unclear. The subtelomere-encoded variable secreted protein (SVSP) family is the largest multigene family in both the *T. annulata* and *T. parva* genomes and has unique gene features, including atypical codon usage, length diversity and high levels of insertions and deletions [25]. Based on previous evidence, the SVSP family may play vital roles in immune evasion and transformation of host cells [1, 25–27], but the mechanisms by which SVSP molecules initiate host cell signaling “hubs” leading to cancer-like phenotypes remain unknown.

Therefore, elucidating the roles of SVSP family members in parasite-host interactions will provide a new opportunity to explore *Theileria*-induced cell transformation. In the present study, SVSP455, a member of the SVSP multigene family from *T. annulata*, was confirmed to directly interact with bovine heat shock protein 60 (HSP60) protein and to modify the mitochondrial apoptotic pathway of host cells.

## Methods

### Cell culture

HEK293T- and *T. annulata* schizont-infected cells were provided by the Vector and Vector Borne Disease team, Lanzhou Veterinary Research Institute (LVRI), China. HEK293T cells were cultured in Dulbecco's Modified Eagle Medium (Gibco™, Thermo Fisher Scientific, Waltham, MA, USA) supplemented with 10% fetal bovine serum (FBS) (Gibco™, Thermo Fisher Scientific) at 37 °C in the presence of 5% CO_2_. *Theileria annulata*-infected cells were maintained in RPMI 1640 medium (Biological Industries, Kibbutz Beit Haemek, Israel) containing 10% FBS (Biological Industries, Kibbutz Beit Haemek, Israel).

### Quantitative real-time PCR

Total RNA was extracted from *T. annulata* at three life-cycle stages (schizont, sporozoite and merozoite) using an RNeasy Mini Kit (QIAGEN, Hilden, Germany) according to the user manual. The complementary DNA (cDNA) templates were then synthesized using the PrimeScript™ RT Reagent Kit with gDNA Eraser (Perfect Real Time; TaKaRa, Bio Inc., Kusatsu, Shi, Japan) following evaluation of the concentration and quality of RNA. The specific primers for the target genes were designed according to the sequences derived from the NCBI database. The levels of the β-actin transcript in *T. annulata* and bovine cells were used for normalization. All primer sequences used in the present study are shown in Additional file 1: Table S1. Quantitative real-time PCR assays were performed in the Stratagene MX3005P thermocycler (Agilent Technologies, Santa Clara, CA, USA) and using the TB Green® Premix Ex Taq™ kit (Tli RNaseH Plus; TaKaRa Bio Inc.) according to the user manual. The relative transcript levels of the target genes were calculated using the comparative cycle threshold (2^−ΔΔ*CT*^) method.

### Western blotting analysis

Cells were lysed with RIPA lysis buffer (Boster Bio, Pleasanton, CA, USA; #AR0102) containing protease inhibitors (Roche, Basel, Switzerland; #4693132001) and a phosphatase inhibitor cocktail (Roche, Basel, Switzerland, #4906845001) for 30 min on ice. The supernatants of the cells were then collected by centrifugation at 15,000 *g* for 15 min at 4 °C, and the concentration of the total proteins was determined using a Pierce™ BCA Protein Assay Kit (Thermo Fisher Scientific; #23225). The protein samples were separated in sodium dodecyl sulfate-polyacrylamide electrophoresis gels and transferred to PVDF membranes (MilliporeSigma, Burlington, MA, USA). Membranes were first blocked with blocking buffer (Tris-buffered saline, pH 7.4, containing 0.05% Tween-20 and 5% bovine serum albumin [BSA]) for 2 h at RT and then incubated with the relevant primary antibodies at 4 °C overnight. Incubations with an horse radish peroxidase (HRP)-conjugated anti-rabbit or anti-mouse secondary antibody (Abcam, Cambridge, UK; #ab6721 or #ab6789) were performed for 1 h at room temperature (RT). The proteins levels were determined using SuperSignal™ West Pico PLUS Chemiluminescence Substrate (Thermo Fisher Scientific; #34577).

### Subcellular localization of the SVSP455 protein in *T. annulata*-infected cells

The protein coding sequence (CDS) of SVSP455 was determined from *Theileria annulata*-infected cells by PCR assay, and the sequence of SVSP455 was analyzed by informatics assays. The amino acids of SVSP455 from 21 to 421 residues were used as the target sequences to prepare the antibody against SVSP455. The recombinant expression plasmid SVSP455-pET30a was then successfully obtained after sequencing and enzyme digestion identification. Finally, the recombinant protein SVSP455 was expressed in *Escherichia coli* and was purified via Ni–NTA Agarose. After determining the concentration and reactivity of the purified protein, 200 mg of the protein was used to immunize rabbits, with three injections at 14-day intervals. The sera against SVSP455 protein derived from rabbit immunization were collected and purified using the NAb™ Protein A/G Spin Kit (Thermo Fisher Scientific; #89980). The antibody against TaSP served as the positive control. Subcellular distribution of SVSP455 in *T. annulata*-infected cells was determined by confocal microscopy. In brief, for the confocal microscopy experiment, *T. annulata*-infected cells were seeded onto glass slides in 12-well cell culture plates at an initial density of 3.0 × 10^6^ cells/ml and incubated at 37 °C with 5% CO_2_ for 24 h. The cells were then fixed with 4% paraformaldehyde at RT for 30 min, washed with phosphate buffered saline (PBS) and permeabilized with 0.5% Triton X-100 in PBS for 15 min at RT. After three washes with PBS, the cells were blocked with 3% BSA for 1 h at RT, then incubated with an anti-SVSP455 antibody at a dilution of 1:100 in PBS containing 3% BSA at 4 °C overnight. Next, 500 μl of a donkey anti-rabbit secondary antibody conjugated to Alexa Fluor 488 (Life Technologies, Thermo Fisher Scientific; #A21206) was added and incubated for 37 °C at RT. Finally, the nucleus and cytoskeleton of the cells were stained with Hoechst 33342 and Alexa Fluro™ 594-conjugated phalloidin (Life Technologies, Thermo Fisher Scientific; #H3570 and #A13281), respectively. More than 100 random cells per slide were visualized using a confocal microscope (TCA; Leica Microsystems, Wetzlar, Germany) with a 63× oil objective, and the most representative images from each sample were used for presentation.

### Construction and bioinformatic analysis of the bait plasmid

A fragment of SVSP455 was amplified from the cDNA of *T. annulata* with specific primers designed based on the reference sequence (Accession No: XM_950455.1 or Piroplasm DB: TA05545) using PCR. The primer sequences used to detect SVSP455 were SVSP455-F (5′-CCGATTCCGTATAAATGTGTAACATAT-3′) and SVPS455-R (5′- TGCACTGCAGTGCATGTTTTATAGGTCGCTTTAAT-3′); the underlined sequences indicate restriction enzyme sites (EcoRI and PstI). The PCR products were purified using a Cycle-Pure kit (OME Bio-Tek, Norcross, GA, USA). Both the pGBKT7 vector and purified PCR products of SVSP455 were digested with the enzymes *Eco*RI and *Pst*I (Thermo Fisher Scientific; #FD0274 and #FD0614), and then the digested products of the vector and SVSP455 were ligated using T4 DNA ligase (New England Biolabs, Ipswich, MA, USA; #M0202S) according to the manufacturer’s instructions. Finally, the recombinant bait plasmid (SVSP455-pGBKT7) was sequenced (Sangon Biotech, Shanghai, China).

### Assessment of the auto-activation and toxicity of the bait plasmid

The empty pGBKT7 plasmid and the constructed bait plasmid were transformed into Y2H Gold competent cells using the Quick & Easy Yeast Transformation Mix (Clontech Laboratories, Mountain View, CA, USA). The transformants were then incubated on agarose plates containing various components, including SDO, SDO/X and SDO/X/A (see Abbreviation list for description of agar plates), at 30 °C for 3–5 days. When the colonies growing on SDO and SDO/X agar plates acquired a white or pale color, the bait plasmid was not auto-activated. No colonies grew on the SDO/X/A ar plates. Moreover, SVSP455-pGBKT7 was considered toxic if the sizes of colonies cultured on the SDO and SDO/X ar plates were significantly smaller than those transformed with the pGBKT7 plasmid. The yeast two-hybrid (Y2H) system used to screen the interacting proteins could be used only when the bait plasmid was neither auto-activated nor toxic.

### Y2H screening

The Y2H screening assay was used to determine the host cell proteins that interacted with SVSP455. First, the recombinant SVSP455-pGBKT7 plasmid and prey plasmid (the cDNA library of bovine B cells) [28] were co-transformed into Y2H Gold competent cells using Yeastmaker™ Yeast Transformation System 2 (Clontech Laboratories) according to the manufacturer’s recommendations. The transformed competent cells were then cultured on DDO/X/A ar plates at 30 °C for 3–5 days, and the blue colonies from the DDO/X/A ar plates were picked and cultured on QDO/X/A agar plates for a further 3–5 days at 30 °C. Finally, the blue colonies were plated once again on the QDO/X/A agar plates to reduce the number of false-positive clones (see Abbreviation list for description of ar plates). At the same time, both the negative control (pDT7-T and pGBKT7-Lam plasmids) and positive control (pDT7-T and pGBKT7-53 plasmids) were co-transformed into Y2H Gold competent cells and cultured on DDO and DDO/X/A agar plates at 30 °C for 3–5 days.

### Rescue and analysis of prey plasmids

The blue colonies grown on QDO/X/A agar plates were preliminarily detected with PCR using the Matchmaker™ Insert Check PCR Mix (Clontech Laboratories). The potential prey plasmids were then isolated from the identified blue colonies using the Easy Yeast Plasmid Isolation Kit (Clontech Laboratories). A 3-μl aliquot of prey plasmids extracted from yeast cells was transformed into competent cells (*E. coli* DH5α) for plasmid rescue and sequencing. The gene fragments were analyzed with BLAST from the US National Center for Biotechnology Information (NCBI) to identify the host genes. The biological processes and structures of the identified genes were also analyzed using the UniProt database (http://www.uniprot.org/) and SMART server (http://smart.embl-heidelberg.de/).

### Expression and subcellular colocalization analysis of SVSP455 and its prey proteins

The recombinant plasmid (pcDNA3.1-SVSP455-Myc) was constructed by cloning SVSP455-Myc into the pcDNA3.1 vector after *Bam*HI/*Xho*I digestion (Thermo Fisher Scientific; #FD0054 and #FD0694, respectively). The recombinant p3×Flag-CMV-prey genes plasmids were constructed by cloning the fragments of the prey genes into the p3×Flag-CMV vector at the *Hin*dIII/*Xba*I sites (Thermo Fisher Scientific; #FD0504 and #FD0684, respectively). The expression and subcellular localization of SVSP455 and its interacting proteins were examined by confocal microscopy. HEK293T cells were cultured on glass slides in the 6-well culture plates at an initial density of 5 × 10^5^ cells/ml, following which the constructed plasmids carrying SVSP455 and its prey genes were transfected or cotransfected into the cells using Lipofectamine™ 3000 transfection reagent (Thermo Fisher Scientific; #L3000015) when the confluence rate of the cells reached 70–90%. After 24 h of transfection, the cells on the glass slides were washed, fixed, permeabilized and then blocked using the procedures described above. The cells were then stained with a rabbit anti-Myc tag monoclonal antibody (mAb) (Cell Signaling Technology, Danvers, MA, USA; #2278S) or an anti-Flag tag mAb derived from mouse (Sigma-Aldrich, St. Louis, MO, USA; #F1804) overnight at 4 °C. After the cells were washed with PBS three times, they were incubated with Alexa Fluor 594-conjugated donkey anti-rabbit or Alexa Fluor 488-conjugated goat anti-mouse secondary antibodies (Life Technologies, Thermo Fisher Scientific; #A21207 and #A11029, respectively) at a dilution of 1:1000 in PBS containing 3% BSA at RT for 1 h. Hoechst 33342 (Life Technologies, Thermo Fisher Scientific; #H3570) was used to label the nuclei followed by five washes with PBS. A confocal microscope (TCS; Leica Microsystems) was used to visualize the fluorescence with a 63× oil objective.

### Co-immunoprecipitation assay

A co-immunoprecipitation (Co-IP) assay was performed to identify whether SVSP455 binds to the identified prey proteins. First, 2 × 10^6^ HEK293T cells per dish were seeded into 10-cm-diameter cell culture dishes. Both the pcDNA3.1-SVSP455-Myc recombinant plasmid (10 µg) and p3×Flag-CMV-prey plasmids (10 µg) were cotransfected into the cells when the cell confluence rate was 70–90%. After 48 h of culture, the cells were collected and washed with PB, followed by lysing with 600 μl of IP/Lysis buffer containing phosphatase inhibitors (Roche; #4906845001) and protease inhibitors (Roche; #4693132001) on ice for 30 min. Cell lysates were centrifuged at 16,000 *g* for 10 min at 4 °C, and the supernatant was collected and used in the Co-IP experiment, which was performed with a mouse anti-Flag tag monoclonal antibody using a Pierce™ Co-Immunoprecipitation Kit (Thermo Fisher Scientific; #26149). At the same time, the empty plasmids (pcDNA3.1 and p3×FLAG-CMV) were cotransfected into HEK293T cells and used as the negative control. The eluted samples after Co-IP were used for western blotting. The target proteins were detected using rabbit anti-Myc tag and anti-Flag tag antibodies.

### Bimolecular fluorescence complementation assay

A bimolecular fluorescence complementation (BiFC) assay was conducted to investigate the interactions between SVSP455 and its prey proteins in cells [29]. SVSP455 and its potential prey genes were cloned into the pBiFC-VN173 vector at the HindIII/SalI sites (Thermo Fisher Scientific; #FD0504 and #FD0644, respectively). At the same time, the recombinant plasmids (pBiFC-VC155-SVSP455 and pBiFC-VC155-prey genes) were constructed by cloning the target fragments into the pBiFC-VC155 vector followed by digestion with *Sal*I/*Kpn*I (Thermo Fisher Scientific; #FD0644 and #FD0524, respectively). The pairs of pBiFC-VC155-SVSP455/pBiFC-VN173-prey genes and pBiFC-VC155-prey genes/pBiFC-VN173-SVSP455 were cotransfected into HEK293T cells. Untransfected cells served as the control. At 24 h after transfection, the green signals of BiFC and their interaction with SVSP455 and its prey proteins were visualized using confocal microscopy. For the confocal experiments, HEK293T cells were stained with a rabbit anti-HA tag mAb (Cell Signaling Technology; #3724S) or mouse anti-Flag tag mAb (Sigma-Aldrich; #F1804) followed by fixation, permeabilization and blocking of the cells at 4 °C overnight. The cells were then labeled with 1 µg/ml donkey anti-rabbit IgG (H + L) antibody conjugated to Alexa Fluor® 594 (Life Technologies, Thermo Fisher Scientific; #A21207) or Alexa Fluor® 594-conjugated goat anti-mouse antibody (Life Technologies, Thermo Fisher Scientific; #A11005) at RT for 1 h followed by three washes with PBS. The nuclei were stained with Hoechst 33342 (Life Technologies, Thermo Fisher Scientific; #H3570).

### Flow cytometry

HEK293T cells were cultured in 6-well cell culture plates at an initial density of 5 × 10^5^ cells/ml. The constructed BiFC plasmids pBiFC-VC155-SVSP455, pBiFC-VN173-prey genes, pBiFC-VC155-prey genes and pBiFC-VN173-SVSP455 were transfected individually into the cells. Moreover, the pBiFC-VC155-SVSP455/pBiFC-VN173-prey genes and pBiFC-VC155-prey genes/pBiFC-VN173-SVSP455 pairs were cotransfected into the cells. At 48 h after transfection, the cells were harvested after digestion with trypsin–EDTA (0.25%; Gibco™, Thermo Fisher Scientific) and centrifugation at 800 rpm for 5 min. The cells were then resuspended in cold PBS, and the mean fluorescence intensity (MFI) of the transfected cells was determined using an Accuri™ C6 Plus Flow cytometer (BD, Franklin Lakes, NJ, USA).

### Subcellular colocalization analysis of SVSP455 and its interacting proteins in *T. annulata*-infected cells

Confocal microscopy was used to further identify the interaction between the native SVSP455 and its prey proteins in *T. annulata*-infected cells. The mouse polyclonal antibodies against prey proteins were prepared and purified using the NAb™ Protein A/G Spin Kit (Thermo Fisher Scientific; #89980) (data not shown). For the confocal experiments, *T. annulata*-infected cells were cultured on glass slides in 12-well cell culture plates at an initial density of 5 × 10^5^ cells/ml. After 24 h of culture, the cells were labeled with rabbit anti-SVSP455 polyclonal antibodies and mouse anti-prey proteins at a dilution of 1:200 at 4 °C overnight, followed by fixation, permeabilization and blocking. Cells were stained with a Alexa Fluor® 594-conjugated donkey anti-rabbit secondary antibody (Life Technologies, Thermo Fisher Scientific; #A21207) and Alexa Fluor® 488-conjugated goat anti-mouse (Life Technologies, Thermo Fisher Scientific; #A11029) secondary antibody at RT for 1 h. After five washes with PBS, Hoechst 33342 was applied at a dilution of 1:2000 (Life Technologies, Thermo Fisher Scientific; #H3570) to stain the nuclei and incubated for 15 min at RT. The images were acquired using a confocal microscope (TCS; Leica Microsystems) with a 63× oil objective.

### Transfection of *T. annulata*-infected cells

The *T. annulata*-infected cells were cultured in 25 cm^2^-cell culture flasks at an initial density of 0.5 × 10^6^ cells/ml. The recombinant plasmid-pcDNA3.1-SVSP455-Myc (5 µg) was transfected into *T. annulata*-infected cells using an Amaxa® Human T-Cell Nucleofector® Kit (Lonza Group AG, Basel, Switzerland; #VPA-1002) and Nucleofector® Program V-024 (Lonza Group AG) according to the manufacturer’s instructions. The transfected cells were then treated with the antiparasitic drug buparvaquone (Bup) (MedChemExpress, Monmouth Junction, NJ, USA; #HY-17581) at a concentration 200 ng/ml for 48 h. At the same time, the untransfected cells and the cells treated with Bup served as the controls. The cell samples were then collected to identify the target molecules using qPCR and western blotting assays. The *T. annulata*-infected cells were also transfected with 200 nM short interfering RNA (siRNA) against the prey genes (Additional file 2: Table S2). At 48 h after transfection, the cells were examined to detect changes in the expression of the related molecules using qPCR and western blotting.

### Data and statistical analysis

The GraphPad PRISM 9 software package (GraphPad Software Inc., San Diego, CA, USA) was used for statistical analyses. In all figures, the significance of differences between groups was determined using the unpaired two-tailed Student’s t-tests. The variance of each group was estimated by calculating the standard error of the mean. Experiments were repeated independently at least 3 times, and representative results are shown in all figures.

## Results

### SVSP455 expression pattern in *T. annulata*-infected cells

**Fig. 1:**
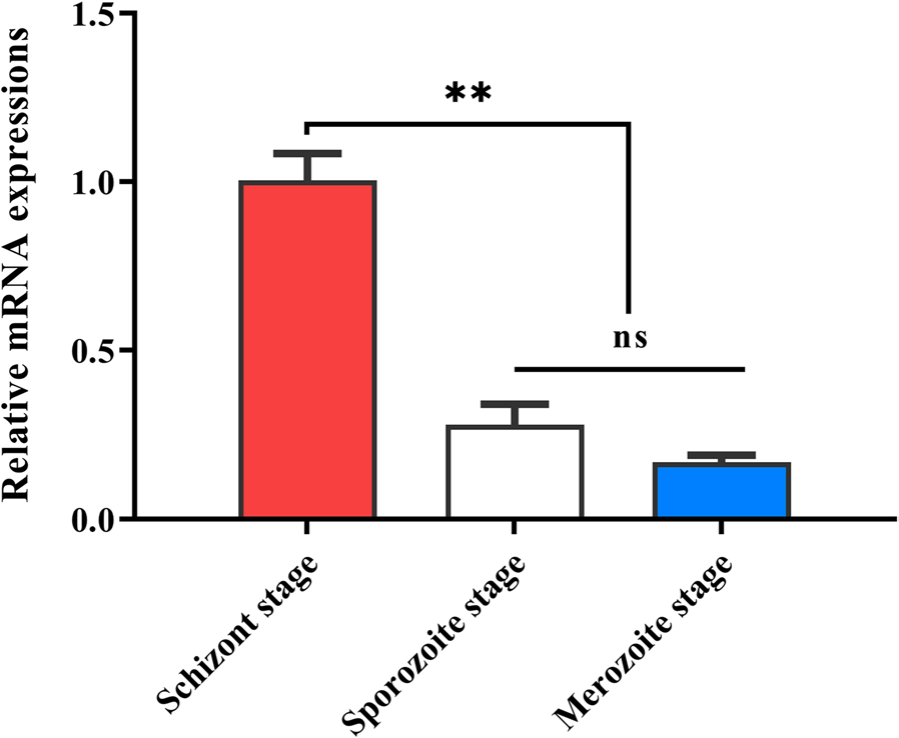
Analysis of SVSP455 mRNA levels in different life-cycle stages of *Theileria annulata*. β-actin mRNA was used for normalization. Asterisks indicate a significant difference at **P < 0.01; ns indicates difference was not significant (*P* > 0.05). *mRNA,* Messenger RNA, *SVSP455,* subtelomere-encoded variable secreted protein

Based on the qPCR results (Fig. 1), SVSP455 mRNA was mainly expressed in the *T. annulata* schizont stage, consistent with the findings of previous studies [30, 31].

### Subcellular localization of SVSP455 in *T. annulata* schizont-infected cells

**Fig. 2:**
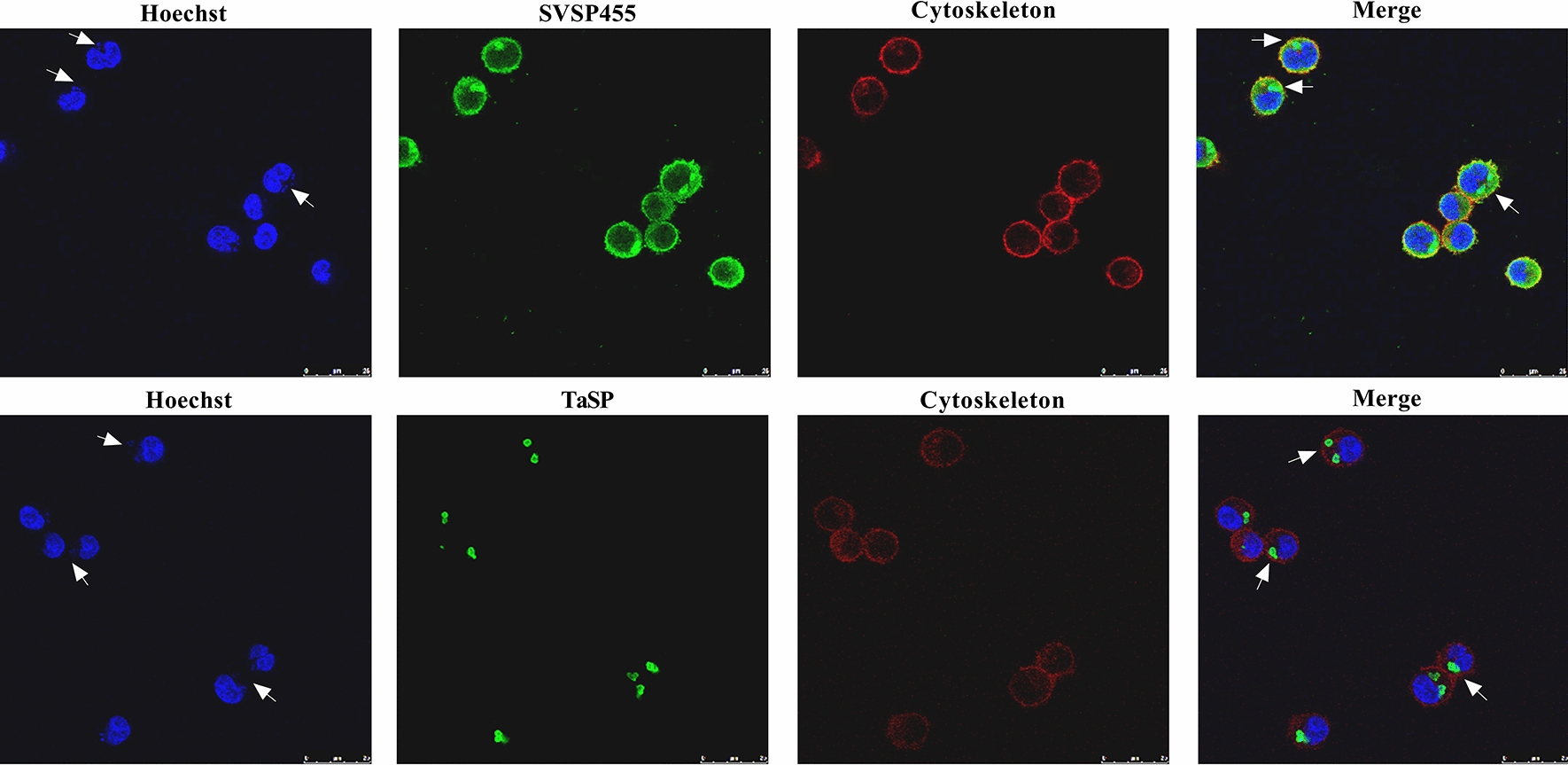
Subcellular localization of SVSP455 in *T. annulata*-transformed cells analyzed using confocal microscopy. SVSP455 and TaSP proteins were probed with their specific polyclonal antibodies derived from rabbits. The white arrows in the confocal pictures pointed to the *T. annulata* schizont. Scale bar: 25 μm. *TaSP,* *Theileria annulata* surface protein

Confocal microscopy results indicated that SVSP455 was mainly distributed on the surface of *T. annulata* schizonts and in the cytoplasm of *T. annulata*-infected cells (Fig. 2).

### Bioinformatic identification of the bait plasmid

**Fig. 3:**
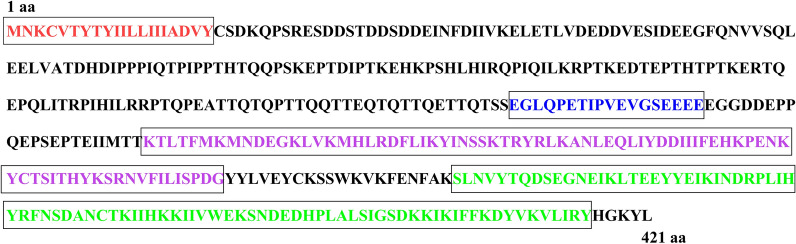
Schematic showing the structural features of SVSP455 identified by bioinformatic analysis. The signal peptide (1–20 aa) is shown in red; the Tash-PEST motif (193–210 aa), in blue; internal repeat 1 (RPT1) domain (232–310 aa), in purple; the FAINT domain (332–416 aa), in green. *aa* Amino acid

A fragment of the SVSP455 gene was successfully amplified from the complementary DNA (cDNA) of *T. annulata*-transformed cells, and the recombinant bait plasmid (SVSP455-pGBKT7) was also constructed after sequencing. The CDS region of SVSP455 is 1263 bp, encoding 421 amino acids (aa), with a predicted molecular weight of 49 kDa. However, the CDS of the reference gene is 1293 bp, encoding 431 aa. The identity of the nucleotide and amino acid sequences of the amplified SVSP455 sequence and the reference gene was 93.9% and 90.1%, respectively. As shown in Fig. 3, SVSP455 contains a signal peptide from amino acids 1 to 20 but no putative glycosylphosphatidylinositol (GPI) anchors, nuclear localization signal sequences (NLSs) or transmembrane domains based on the predictions from SignalP-5.0 (http://www.cbs.dtu.dk/services/SignalP-5.0/), the PredGPI server (http://gpcr.biocomp.unibo.it/predgpi/pred.htm), the NLStradamus (http://www.moseslab.csb.utoronto.ca/NLStradamus/) and TMHMM server (http://www.cbs.dtu.dk/services/TMHMM/). Moreover, the SVSP455 protein was predicted to contain a Tash-PEST motif from 193 to 210 aa, an internal repeat 1 (RPT1) domain ranging from 232 to 310 residues and a FAINT domain between 331 and 416 aa, as analyzed using the SMART server (http://smart.embl-heidelberg.de/) and Pfam.

### Y2H screening and analysis of the prey proteins

**Fig. 4:**
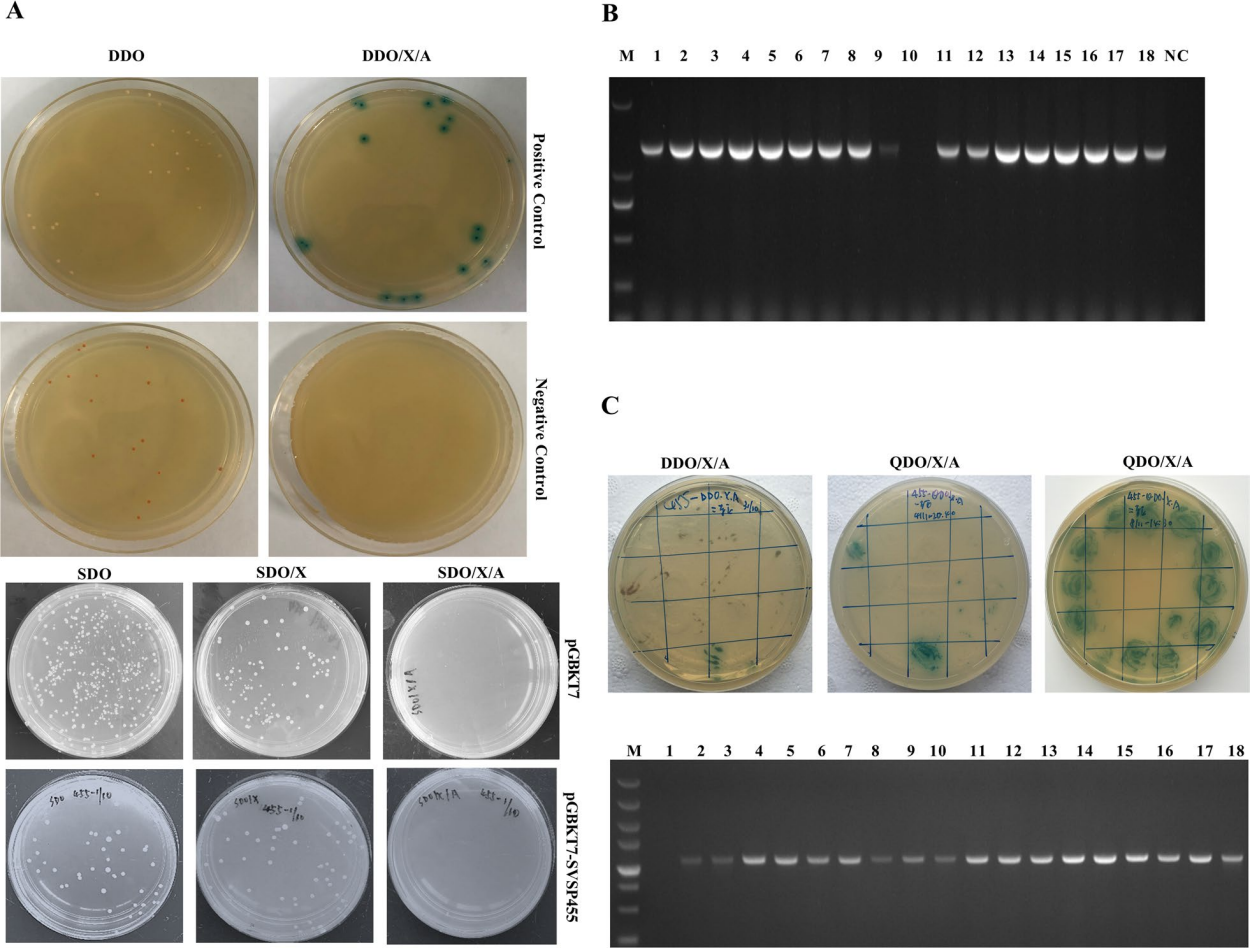
Host cell proteins interacting with the SVSP455 protein were screened using the Y2H system. **A** Toxicity and autoactivation analysis of pGBKT7-SVSP455 in Y2H Gold cells. **B** Determination of the bait plasmid transformation efficiency by PCR assay. The colonies from SDO ar plates were randomly picked and used to in a colony PCR assay in a 50-μl system with specific primers. Lanes: M, DL2,000 DNA marker; 1–18, the numbers of selected colonies grown on SDO plates transfected with the bait plasmid; NC, negative control. **C** Y2H screening and identification of potential host cell proteins interacting with SVSP455. The cotransformants of the bait plasmid pGBKT7-SVSP455 and the prey plasmid cDNA library of bovine B cells were grown on DDO/X/A plates, and the blue colonies from DDO/X/A plates were successively cultured on QDO/X/A plates and screened for putative prey proteins through two rounds of incubation. All blue colonies from QDO/X/A plates were selected and identified by the colony PCR assay based on the pDT7-F/R primers. Lanes: M, DL5000 DNA marker (TaKaRa; #3428A); 1, negative control; 2–18, the numbers of blue colonies from QDO/X/A plates. The positive control (pGBKT7-53 and pDT7-T plasmids) and negative control (pGBKT7-Lam and pDT7-T plasmids) were also cotransformed into Y2H Gold cells and cultured onto DDO and DDO/X/A plates. The empty pGBKT7 plasmid served as the blank control was also transformed into Y2H Gold competent cells and cultured on SDO, SDO/X and SDO/X/A plates. Abbreviations: cDNA, Complementary DNA; DDO, synthetically defined medium without tryptophan and leucine; DDO/X/A, DDO supplemented with X-α-l and aureobasidin A; QDO/X/A, DDO without adenine and histidine, supplemented with X-α-l and aureobasidin A; SDO (SD/-Trp), synthetically defined medium; SDO/X, SDO without tryptophan supplemented with X-α-l SDO/X/A, SDO without tryptophan and leucine supplemented with X-α-l and aureobasidin A; Y2H, yeast two-hybrid

The auto-activation and toxicity features of the bait plasmid were investited before prior to using the Y2H system to screen host cell proteins. As shown in Fig. 4A, colonies containing bait plasmid grown on SDO and SDO/X ar plates were white or pale, indicating that the bait plasmid was not auto-activated. The results of the PCR assay using specific primers showed that the transform efficiency of the bait plasmid was 88.89% (Fig. 4B). The potential proteins interacting with the SVSP455 bait plasmid were screened using the Y2H system and identified through colony PCR (Fig. 4C). The nucleotide sequence of the screened prey plasmid shared 100% identity with the *Bos taurus* HSP60 (accession no: KF690729.1) and matched the CDS region of HSP60 from 1574 to 2383 nucleotides.

### *Theileria annulata* SVSP455 interacts with the bovine HSP60 protein

**Fig. 5:**
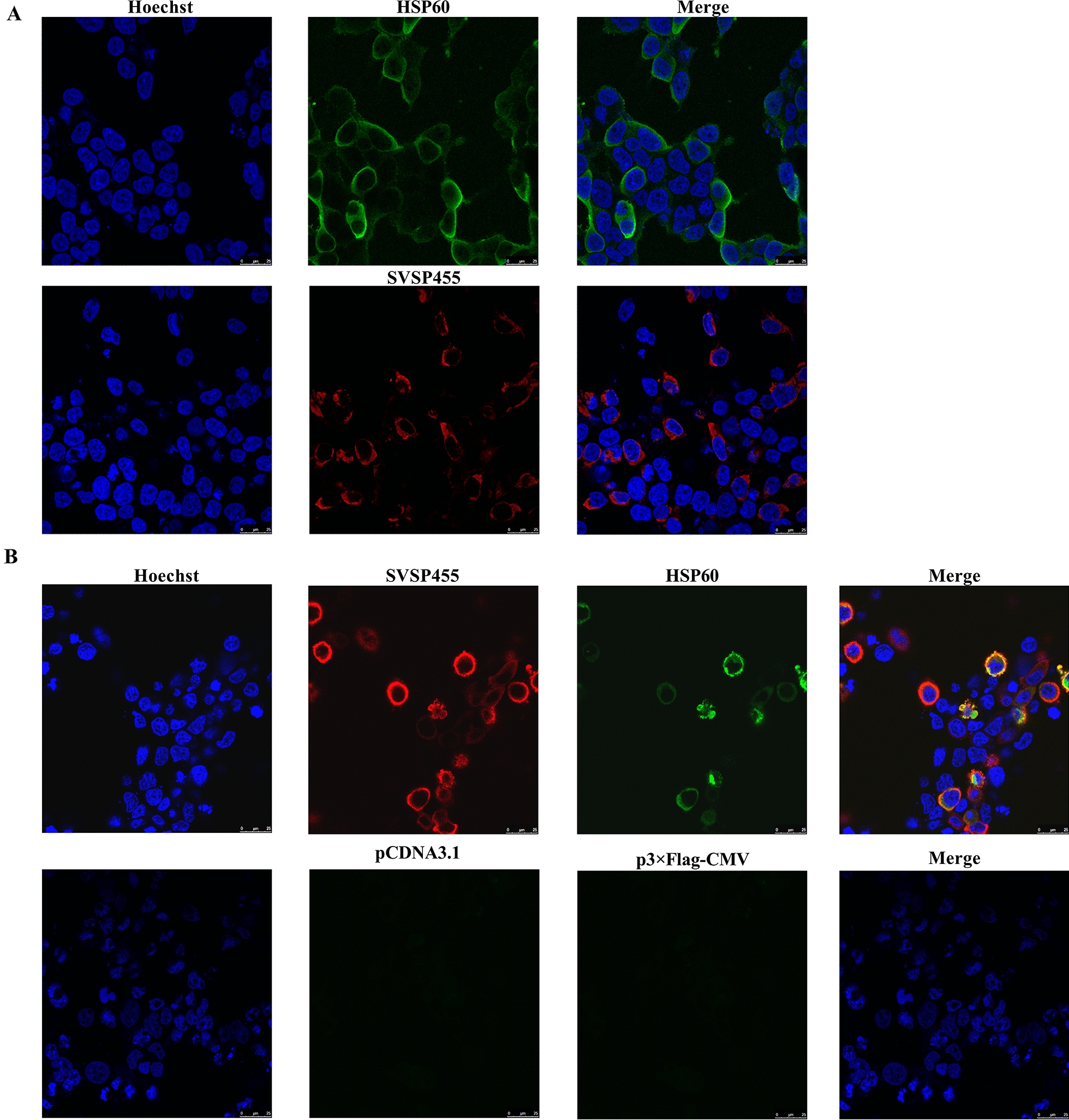
Confocal microscopy identification of SVSP455 and HSP60 expression and their interaction in HEK293T cells. **A** The Myc-tagged SVSP455 (4 μg) and its potential prey protein Flag-tagged HSP60 (4 μg) were individually transfected into HEK293T cells. **B** Both Myc-tagged SVSP455 (2 μg) and Flag-tagged HSP60 (2 μg) were cotransfected into HEK293T cells. The empty plasmids (pcDNA3.1 and p3×FLAG-CMV) were also cotransfected into HEK293T cells and used as the negative control. Scale bar: 25 μm. HSP60: Heat shock protein 60

**Fig. 6:**
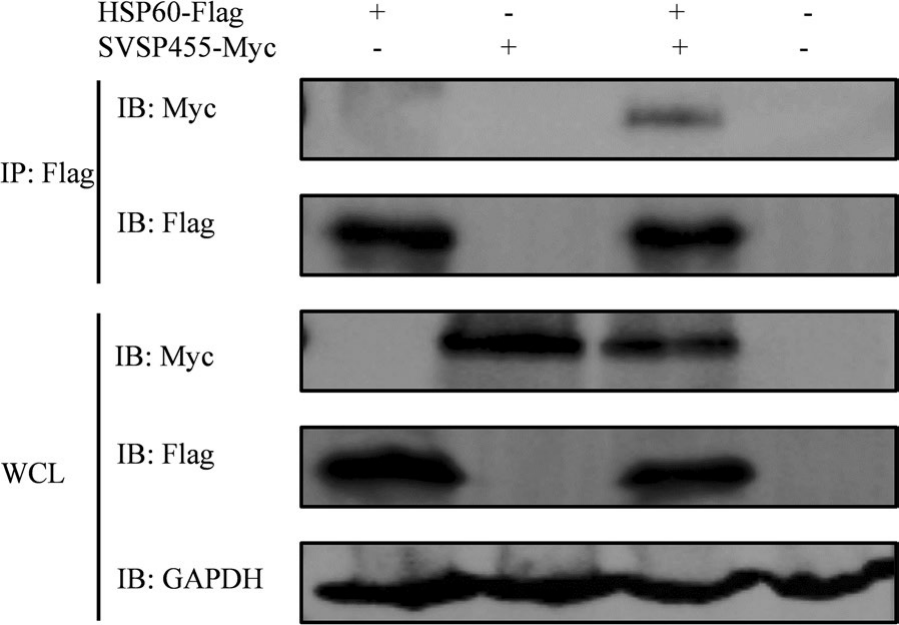
SVSP455 binds to bovine HSP60 in HEK293T cells. Co-IP and IB were used to determine Myc-tagged SVSP455 and its possible interacting host cell protein, Flag-tagged HSP60, in HEK293T cells. The negative control plasmids (pcDNA3.1 and p3×FLAG-CMV) were cotransfected into the cells. GAPDH: Glyceraldehyde-3-phosphate dehydrogenase; IP: immunoblotting; IP: immunoprecipitation;  WCL: whole HEK293T cells

The constructed recombinant plasmids pcDNA3.1-SVSP455-Myc and p3×Flag-CMV-HSP60 were transfected or cotransfected into HEK293T cells. The expressed proteins were detected using confocal microscopy, which showed that Myc-tagged SVSP455 and Flag-tagged HSP60 were expressed alone or together in HEK293T cells (Fig. 5A) and that the two proteins had colocalization characteristics in the cells (Fig. 5B). Moreover, the Co-IP assay showed that SVSP455 interacted with HSP60 when they were over-expressed in the HEK293T cells (Fig. 6).

**Fig. 7:**
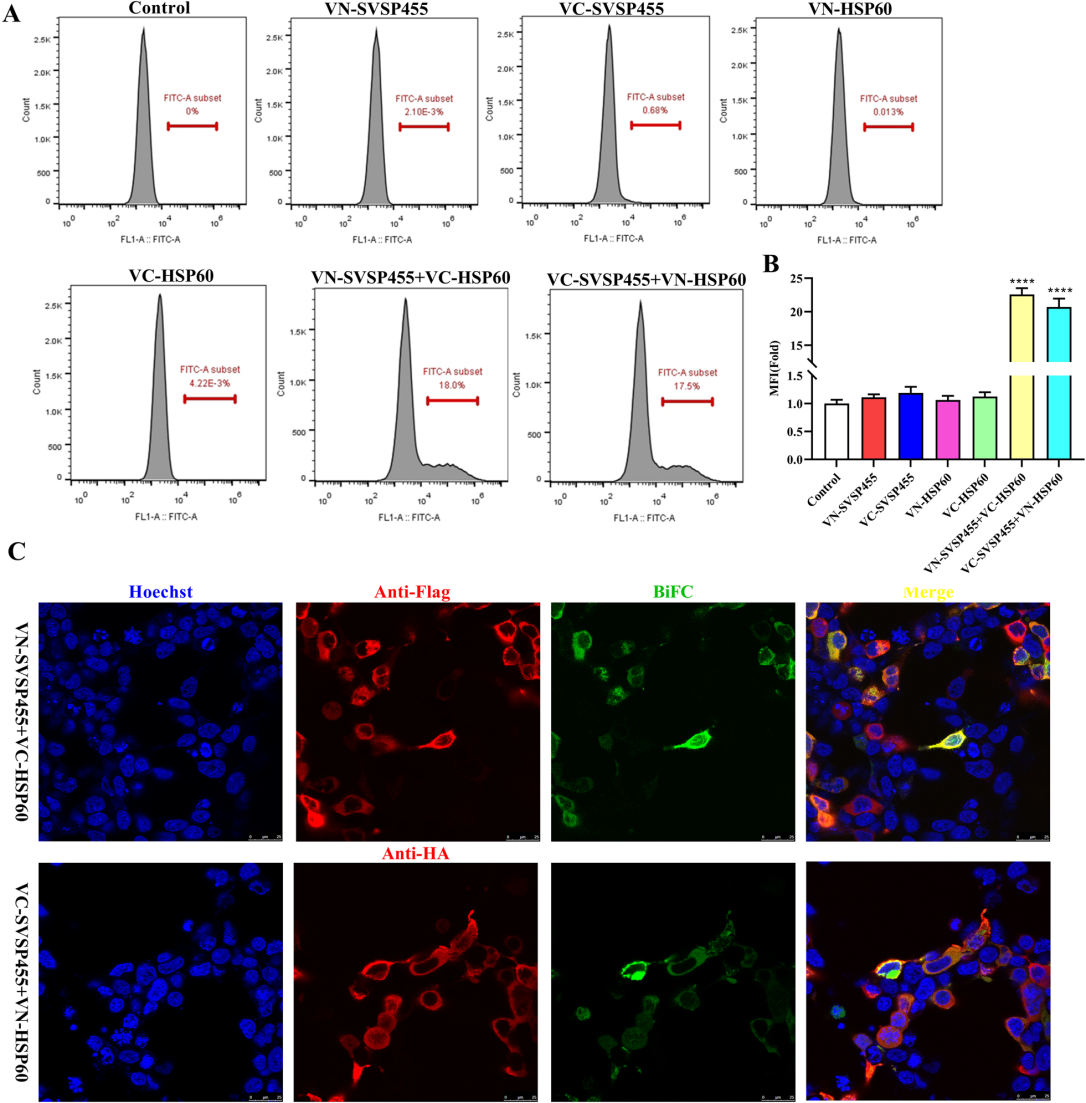
SVSP455 directly interacts with bovine HSP60 in the cell. **A**, **B** The MFI of BiFC green-fluorescent signals was analyzed using a flow cytometry assay and the results are presented as MFI values relative to those of signals of the untransfected cells, which were used as the negative control. **C** The subcellular colocalization of the red signals for VN-SVSP455 and the BiFC green fluorescence signals of interacting pairs was observed using confocal microscopy. Scale bar: 25 μm. Asterisks indicate significant difference at *P < 0.05, **P < 0.01 and ***P < 0.001. Abbreviations: BiFC: Bimolecular fluorescence complementation; MFI: mean fluorescence intensity

The BiFC assay depends on the link between two non-fluorescent complementary fragments of a fluorescent protein, such as green fluorescent protein (GFP) or yellow fluorescent protein (YFP). Only when SVSP455 binds to its partner are these proteins brought in proximity to each other, which leads to a bright fluorescent signal in living cells. The protein–protein interactions for this assay are observed using fluorescence microscopy or flow cytometry [29]. In our study, a BiFC assay was used to further confirm how SVSP455 targets its interacting protein HSP60 in the cell. Flow cytometry detected no fluorescent signals in HEK293T cells transfected with a single plasmid, whereas strong signals were detected by BiFC in the VN-SVSP455/VC-HSP60- or VC-SVSP455/VN-HSP60-transfected cells (Fig. 7A, B). Similar to the flow cytometry results, confocal microscopy also revealed the interaction of SVSP455 and HSP60 from the detected BiFC fluorescent signals in transfected cells (Fig. 7C). The interacting protein pair was distributed in the perinuclear region in HEK293T cells (Fig. 7C).

**Fig. 8:**
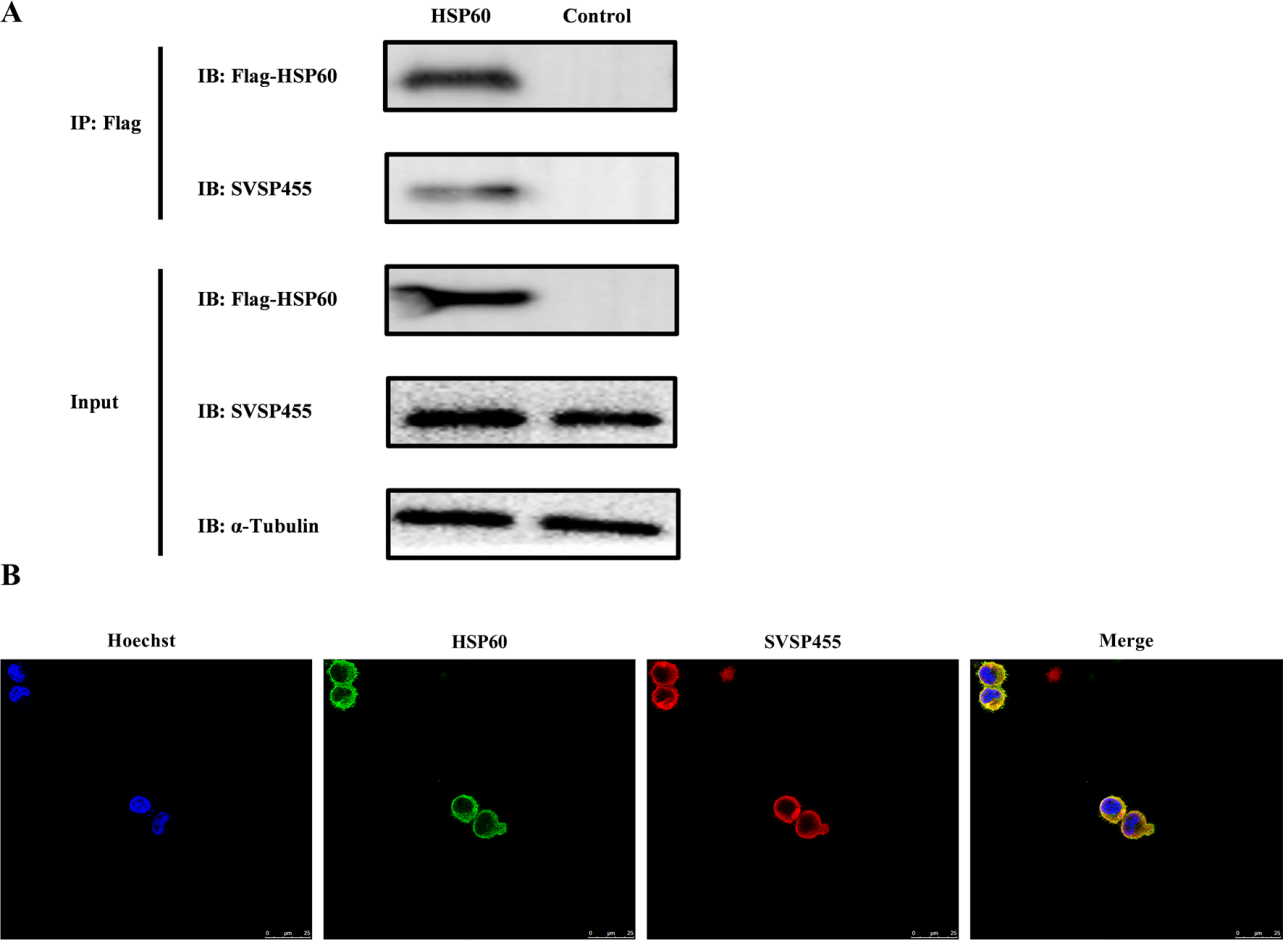
Bovine HSP60 interacts with endogenous SVSP455 in *T. annulata*-infected cells. **A** Flag-tagged HSP60 (5 μg) and the empty plasmid-p3×Flag-CMV ((5 μg), which served as the negative control (NC), were transfected into *T. annulata*-infected cells. **B** Subcellular colocalization analysis of endogenous HSP60 and SVSP455 in *T. annulata*-infected cells. Scale bar: 25 μm

**Fig. 9:**
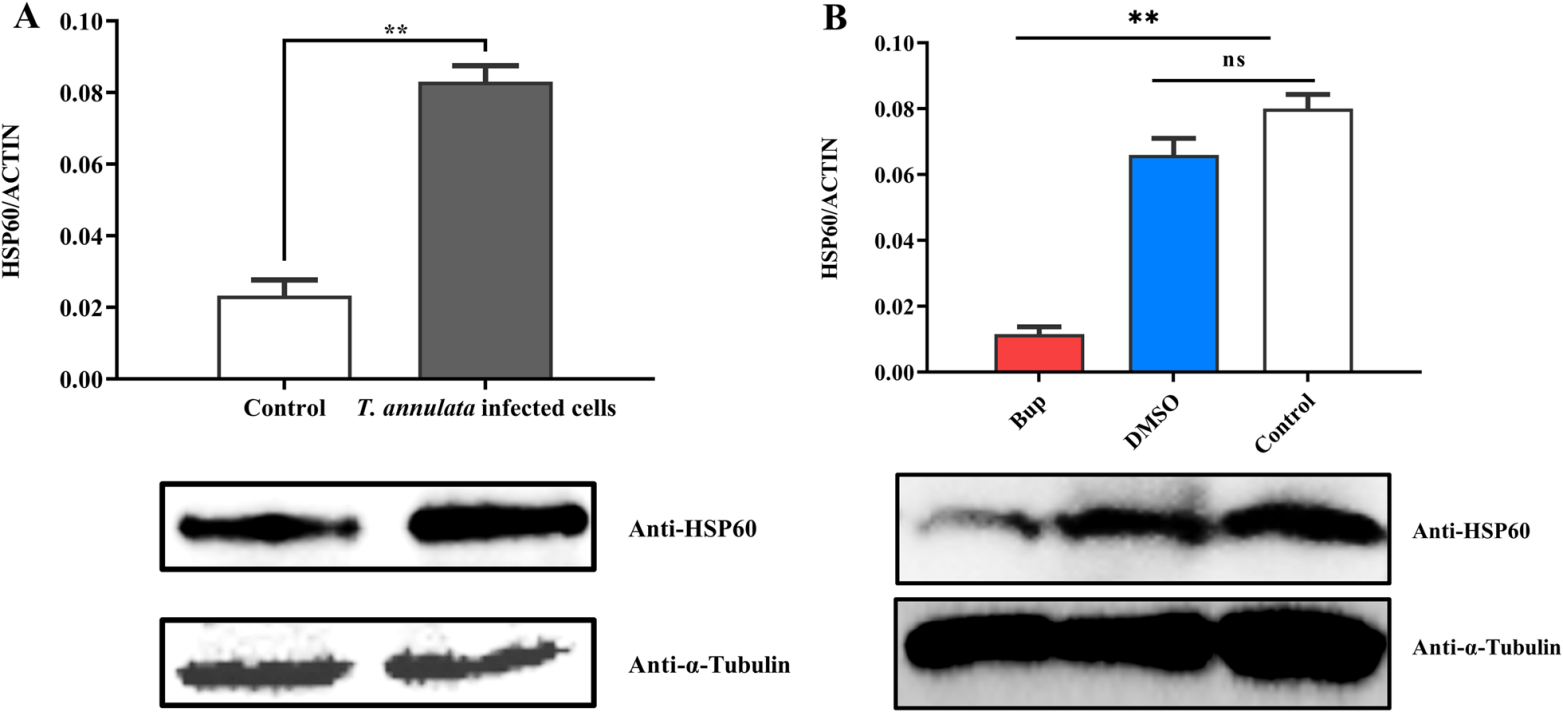
Expression of bovine HSP60 is related to *T. annulata* infection. **A** Analysis of bovine HSP60 expression at the mRNA and protein levels in *T. annulata*-induced cells and noninfected bovine cells. Control: noninfected bovine cells. **B** Comparison of bovine HSP60 expression levels between normal and Bup-treated *T. annulata*-transformed cells using qPCR and immunoblotting. Bup:* T. annulata*-transformed cells upon Bup treatment; Control: *T. annulata*-transformed cells; DMSO: *T. annulata*-transformed cells treated with DMSO. The bovine β-actin mRNA was used for normalization. A mouse anti-α-tubulin monoclonal antibody was used as the loading control. Asterisks indicate significant difference at *P < 0.05,**P < 0.01 and ***P < 0.001. Antiparasitic drug: Buparvaquone (Bup), DMSO: dimethyl sulfoxide; qPCR: quantitative PCR

**Fig. 10:**
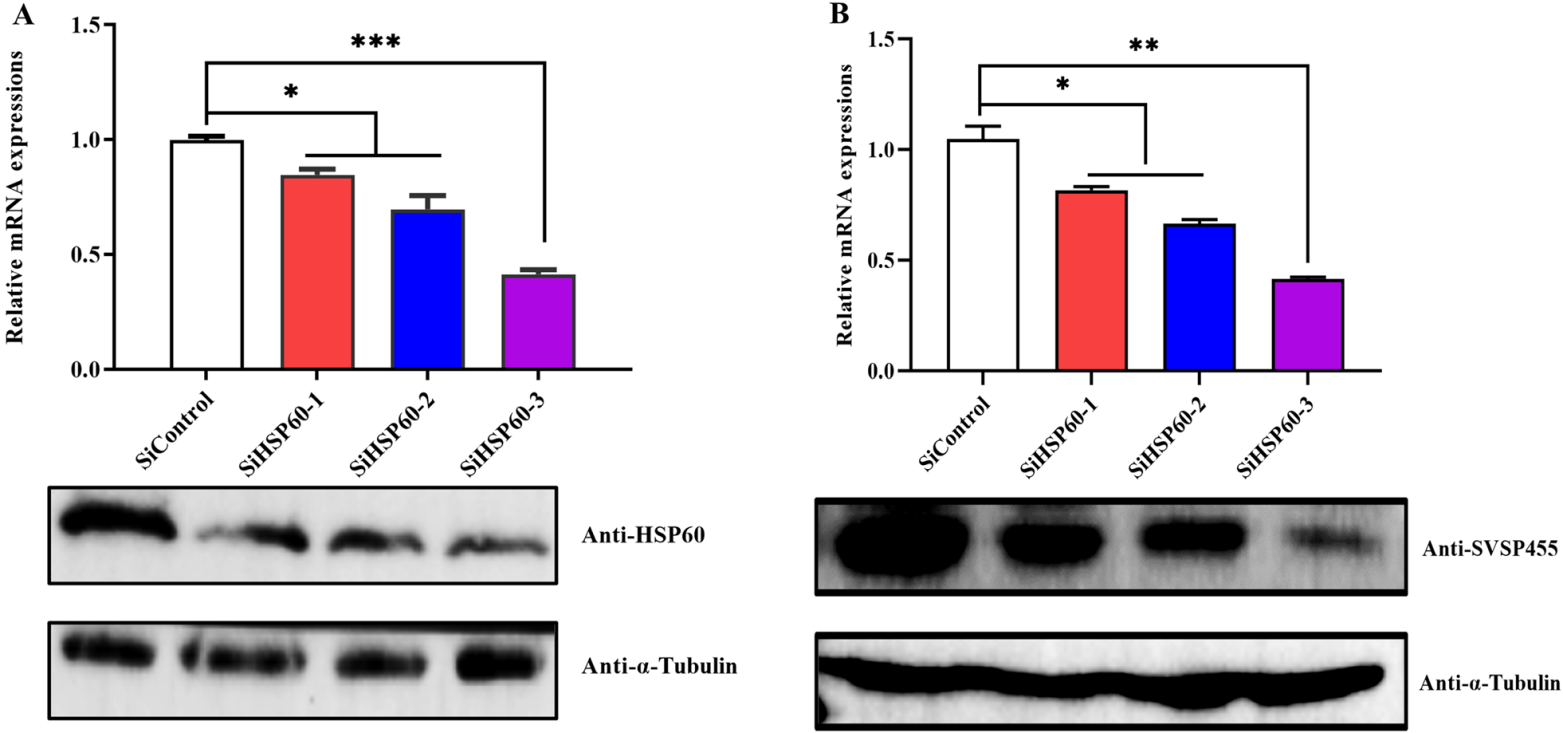
Bovine HSP60 positively regulates *T. annulata* SVSP455. **A** Identification of the efficiency of siRNA-mediated silencing of bovine HSP60 using qPCR and immunoblotting. SiHSP60-1, SiHSP60-2 and SiHSP60-3 are represented as three different siRNAs for targeting bovine HSP60. The bovine β-actin mRNA was used for normalization. **B** SVSP455 expression profiles detected after the knockdown of bovine HSP60 using qPCR and western blotting. *T. annulata* β-actin mRNA was used for normalization. A mouse anti-α-tubulin monoclonal antibody was used as the loading control. Asterisks indicate significance at *P < 0.05, **P < 0.01 and ***P < 0.001. Abbreviations: siRNA: Small interfering RNA; qPCR: quantitative PCR

In Flag-tagged HSP60-transfected *T. annulata*-infected cells, HSP60 also interacted with the endogenous SVSP455 protein, as evidenced by the results of a Co-IP assay (Fig. 8A). The interacting protein pair was distributed on the surface of *T. annulata* schizonts according to the confocal microscopy results (Fig. 8B). As the aim of this study was to investigate the relationship between HSP60 expression and *T. annulata* infection, we looked at the HSP60 mRNA and protein levels in normal and Bup-treated *T. annulata*-infected cells and normal bovine cells. The results revealed elevated levels of host HSP60 in *T. annulata*-infected cells based on qPCR and western blot analyses (Fig. 9). Moreover, SVSP455 expression was noticeably decreased after knockdown of the bovine HSP60 gene (Fig. 10).

### The *T. annulata* SVSP455-HSP60 axis potentially participates in regulating cell apoptosis

**Fig. 11:**
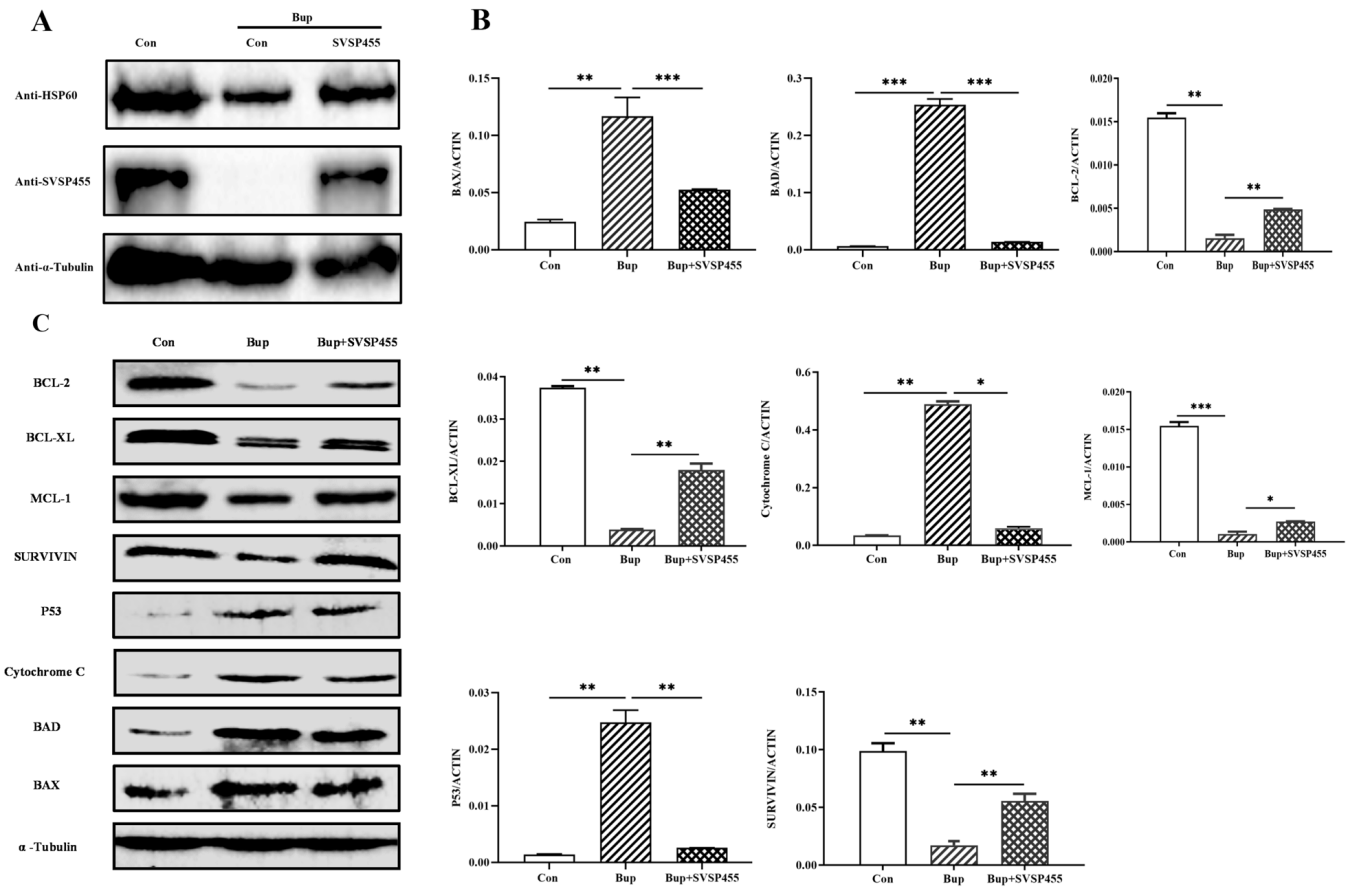
*Theileria annulata* SVSP455 partially rescues HSP60 and host cell apoptosis-related gene expression in *T. annulata*-transformed cells. **A** Myc-tagged SVSP455 partially rescued HSP60 protein levels upon Bup treatment. **B**, **C** *T. annulata* partially rescued expression of host cell genes for mitochondrial apoptosis signaling upon Bup treatment and overexpression SVSP455 + Bup treatment using qPCR (**B**) and immunoblotting (**C**). Bovine β-actin mRNA was used for normalization. A mouse anti-α-tubulin monoclonal antibody was used as the loading control. Con: Control; Bup: Buparvaquone; Bup + SVSP455: *T. annulata*-transformed cells transfected with recombinant plasmid Myc-tagged SVSP455 and treated with Bup; qPCR: quantitative PCR. Asterisks indicate significant difference at *P < 0.05, **P < 0.01 and ***P < 0.001. p Proteins involved in cell apoptosis signaling:  p53, BCL-2, BCL-XL, MCL-1, SURVIVIN, BAX, BAD and Cytochrome *c*

**Fig. 12:**
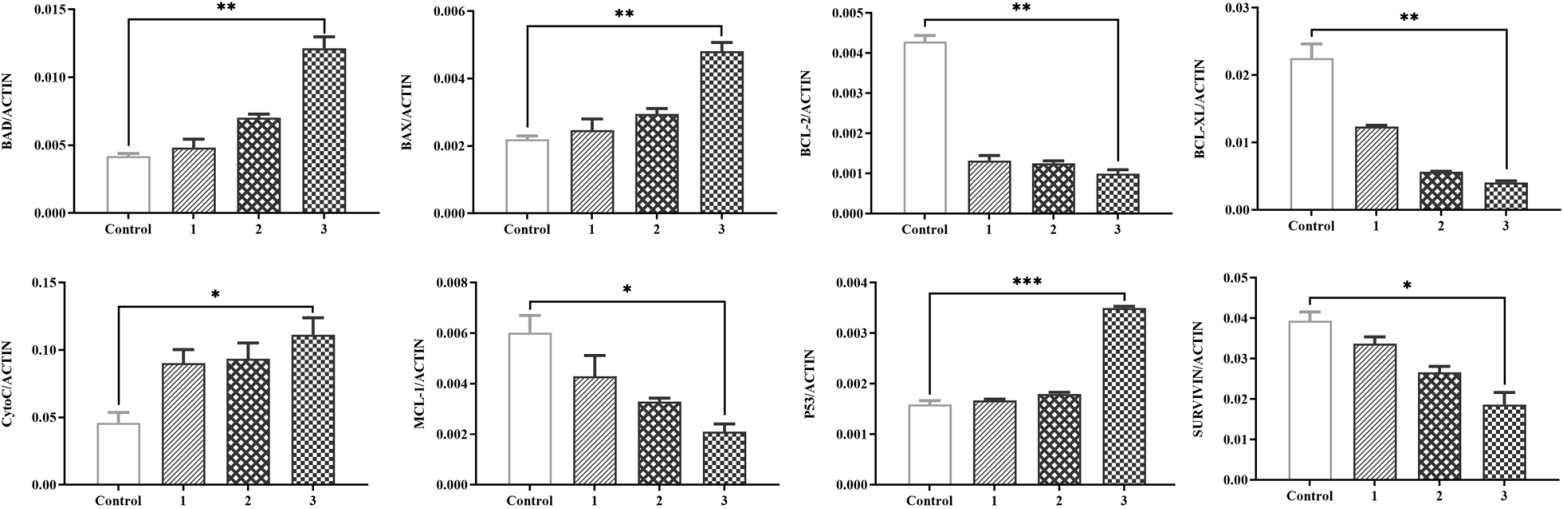
Determination of apoptosis-related gene expression levels after the knockdown of endogenous bovine HSP60 in *T*. *annulata*-transformed cells using qPCR. SiHSP60-1, -2 and -3 are three different SiRNA targeting bovine HSP60. X-axis: 1–3, SiHSP60-1, -2 and -3; Control, SiControl. The bovine β-actin mRNA was used for normalization. Asterisks indicate significant difference at *P < 0.05, **P < 0.01 and ***P < 0.001. SiHSP60: Small interfering RNA against HSP60; qPCR: quantitative PCR; Genes involved in cell apoptosis signaling:  p53, BCL-2, BCL-XL, MCL-1, SURVIVIN, BAX, BAD and cytochrome *c*

In the Bup-treated cells, over-expression of SVSP455 partially rescued the expression levels of host HSP60 (Fig. 11A) and the proteins involved in cell apoptosis signaling, including p53, BCL-2, BCL-XL, MCL-1, SURVIVIN, BAX, BAD and cytochrome* c* (Fig. 11B, C). Detailed information on the antibodies against these proteins is provided in Additional file 3: Table S3. Conversely, siRNA-mediated knockdown of endogenous bovine HSP60 led to an obvious reduction in the mRNA levels of antiapoptotic factors (BCL-2, BCL-XL, MCL-1 and SURVIVIN), whereas proapoptotic genes (BAX, BAD and cytochrome* c*) and p53 were upregulated (Fig. 12). In the present study, the small changes in the expression of pro- and antiapoptotic molecules observed using SiRNA knockdown and overexpression could be attributed to the poor transfection efficiency of both with siRNAs against HSP60 and exogenous SVSP455 in the *T. annulata*-infected cells. Taken together, these data indicate that the interaction of *T. annulata* SVSP455 with HSP60 in *T. annulata*-transformed cells adjusted the expression of pro- and antiapoptotic genes in the mitochondrial apoptosis signaling pathway, which could provide additional opportunities for the proliferation and survival of parasites in the host cells.

## Discussion

Data on the genome and proteome of *T. annulata* are publicly available and helpful for exploring potential parasite proteins that participate in host-parasite interactions [30, 32]. However, the functions of approximately half of these genes remain unclear [1]. During long-term evolution, transforming *Theileria* species (*T. annulata* and *T. parva*) have developed intricate mechanisms to directly manipulate host cell signaling pathways, promoting the uncontrolled propagation of infected cells in a manner similar to cancer cells [33, 34]. Nonetheless, researchers have not yet determined which proteins in the parasites play key roles in cell transformation and their mechanisms.

In the present study, the SVSP455 protein, a member of the SVSP family, served as a target molecule to explore its role in cell transformation. We first found that SVSP455 was predominantly expressed at the *T. annulata* schizont stage, indicating that it may be involved in *T. annulata*-induced pathogenesis. Further, confocal microscopy data indicated that it was mainly localized on the surface of schizonts and in the cytosol of the host cell, suggesting a potential role for this protein in the transformation process. In addition, the results of the bioinformatic analysis showed that the protein contains a signal peptide sequence and is distributed in the cytoplasm of *T. annulata*-infected cells, which further supported the confocal microscopy findings.

The Y2H system was used in our study to search for host proteins that interact with SVSP455. The results of the sequencing and bioinformatics analysis showed that bovine HSP60 was the putative protein interacting with SVSP455. The results from subsequent Co-IP and confocal microscopy assays confirmed the HSP60-SVSP455 interaction. Taken together, these findings indicate that SVSP455 bound to the host HSP60 protein in HEK293T cells. The results of the BiFC assay indicated that the interaction between bovine HSP60 and SVSP455 was direct and that they colocalized in the perinuclear region. More importantly, we also discovered that SVSP455 interacted with endogenous HSP60 in *T. annulata*-infected cells. Moreover, HSP60 expression was significantly upregulated in *T. annulata*-infected cells compared with uninfected cells, and HSP60 was downregulated when parasites were treated with Bup (Figs. 9, 10).

HSP60, which is referred to as a chaperonin, is mainly distributed in the mitochondria of eukaryotes and binds to mitochondrial HSP70 [35]. According to published reports, HSP60 is also located in peripheral blood, on the cell surface and in the cytosol [36, 37]. This protein is not only an antigen of T and B lymphocytes but is also a kind of Toll-like receptor [38, 39], implying that it plays a vital role in the immune system. In the present study, the host HSP60 protein was expressed at a high level in *T. annulata*-transformed cells, consistent with its levels in cancer cells [40, 41], suggesting that it is closely related to cell transformation and carcinogenesis. Studies have indicated that the HSP60 molecule regulates tumor cell apoptosis and strengthens anti-apoptotic effects [42, 43]. In the present study, we discovered that *T. annulata*-infected cells tended to undergo apoptosis after siRNA-mediated knockdown of bovine HSP60. *Theileria* spp. have evolved a crucial strategy to ensures survival and propagation by blocking the apoptosis of infected host cells, including activating the antiapoptotic molecules cFLIP and cIAPs [17, 44], sequestering host p53 [45] and upregulating c-MYC [19]. However, we documented that SVSP455 overexpression partially rescued the expression levels of those proteins involved in the host cell apoptosis signaling pathway to further facilitate parasite survival and proliferation (Fig. 12). Therefore, *T. annulata* SVSP455 may inhibit cell death by hijacking host apoptotic signaling. In addition, HSP60 is a diagnostic and prognostic biomarker for many cancers, such as lung cancer, gastric cancer and leukemia [35, 46]. At the same time, this protein may be a vital drug target for tumor therapy, including in melanoma cells [47, 48]. Therefore, HSP60 is likely to be used as a putative marker or drug target for *T. annulata* infection.

## Conclusion

In the present study, we first demonstrated that the *T. annulata* molecule SVSP455 binds to bovine HSP60 in vitro. Moreover, we found that the interaction of parasite protein SVSP455 with host HSP60 in *T. annulata*-transformed cells regulates the expression profiles of pro- and antiapoptotic genes for the mitochondrial apoptosis signaling pathway. These findings provide pivotal insights into host–pathogen interaction mechanisms and therapeutics that might protect against *Theileria*.

## Supplementary Information

**Additional file 1: Table S1.** Sequence information of the target genes analyzed using qPCR in the present study.

**Additional file 2: Table S2.** The siRNA sequences targeting bovine HSP60.

**Additional file 3: Table S3.** Antibodies targeting the key molecules in the host cell mitochondrial apoptosis signaling pathway.

## Data Availability

The datasets supporting the conclusions of this article are included within the article.

## Abbreviations

BiFC: Bimolecular fluorescence complementation
Co-IP: Co-immunoprecipitation
DDO: Synthetically defined medium without tryptophan and leucine
DDO/X/A: Synthetically defined medium without tryptophan and leucine supplemented with X-α-l and aureobasidin A
HSP60: Heat shock protein 60
QDO/X/A: Synthetically defined medium without tryptophan, leucine, adenine and histidine, supplemented with X-α-l and aureobasidin A
qRT–PCR: Quantitative real-time PCR
SDO (SD/-Trp): Synthetically defined medium without tryptophan
SDO/X (SD/-Trp/X-α-l): Synthetically defined medium without tryptophan supplemented with X-α-l
SD/-Trp/X-α-l/AbA: Synthetically defined medium without tryptophan supplemented with X-α-l
SDO/X/A: Synthetically defined medium without tryptophan and leucine supplemented with X-α-l and aureobasidin A
siRNAi: Small interfering RNA
SVSP: Subtelomere-encoded variable secreted protein
Y2H: Yeast two-hybrid
