# Supplementary material for: Theileria annulata SVSP455 interacts with host HSP60
Source: Parasit Vectors. 2022 Aug 30;15:308. doi: 10.1186/s13071-022-05427-z (PMC9426020; doi:10.1186/s13071-022-05427-z)
Supplement: Supplementary file 1 — Additional file 1: Table S1. Sequence information of the target genes analyzed using qPCR in the present study. [file 13071_2022_5427_MOESM1_ESM.docx]

**Table S1 Sequence information of the target genes analyzed using qPCR in the present study**

| **Gene Name** | **Sequences** | |
| --- | --- | --- |
|  | **Sequence Fwd 5′-3′** | **Sequence Rev 5′-3′** |
| TaSVSP455 | CCTCAGTTAATCACCCGACC | CTCCACCTTCTTCTTCTTCTTC |
| TaACTIN | GAGACCACCTACAACAGCATCATG | CACCTTGATCTTCATGGTGCTGGG |
| BovHSP60 | TACTGGATGCTGCCGGAGTG | CTCCCATGCCACCTCCCATT |
| BovACTIN | CCCTGGAGAAGAGCTACGAG | GAAGGAAGGCTGGAAGAGAG |
| BovBCL-2 | GCCTTCTTTGAGTTCGGA | TTCAGAGACAGCCAGGAGA |
| BovSURVIVIN | CGGCCTGGCAGCTCTACCTC | CCAAATCGGGCTCGTTCTCAGTG |
| BovMCL-1 | GCCATCATGTCGCCCGAAGAG | CCGAGCCTGGACTGTTGTTACTG |
| BovBAX | GGACATTGGACTTCCTTCGAGAG | ATGGTGAGCGAGGCGGTGAG |
| BovCytochrome C | CAGAAGTGTGCCCAGTGCCATAC | GCCTGACCTGTCTTTCGTCCAAAC |
| BovBAD | GGAGGATGAGCGACGAGTTTCAC | TCAACCAGGACTGGAGGAAGCG |
| BovBCL-XL | GAGTCGGATCGCAACTTGGATGG | GGCTCTCGGCTGCTGCATTG |

**Ta:** *Theileria annulata*; **Bov:** Bovine; **ACTIN:** β-ACTIN; **Fwd:** Forward; **Rev:** Reverse.
