# Supplementary material for: Theileria annulata SVSP455 interacts with host HSP60
Source: Parasit Vectors. 2022 Aug 30;15:308. doi: 10.1186/s13071-022-05427-z (PMC9426020; doi:10.1186/s13071-022-05427-z)
Supplement: Supplementary file 2 — Additional file 2: Table S2. The siRNA sequences targeting bovine HSP60. [file 13071_2022_5427_MOESM2_ESM.docx]

**Table S2 The siRNA sequences targeting bovine HSP60**

| **Name** | **Sequences** | |
| --- | --- | --- |
|  | **Sense (5'-3')** | **Antisense (5'-3')** |
| **SiHSP60-1** | GCCGAUGCUGUAGCCGUUATT | UAACGGCUACAGCAUCGGCTT |
| **SiHSP60-2** | GCUCAGGUUGCUACAAUUUTT | AAAUUGUAGCAACCUGAGCTT |
| **SiHSP60-3** | CCAGCCUUGGAGUCAAUAATT | UUAUUGACUCCAAGGCUGGTT |
| **SiControl** | UUCUCCGAACGUGUCACGUTT | ACGUGACACGUUCGGAGAATT |
