# Supplementary material for: Theileria annulata SVSP455 interacts with host HSP60
Source: Parasit Vectors. 2022 Aug 30;15:308. doi: 10.1186/s13071-022-05427-z (PMC9426020; doi:10.1186/s13071-022-05427-z)
Supplement: Supplementary file 3 — Additional file 3: Table S3. Antibodies targeting the key molecules in the host cell mitochondrial apoptosis signaling pathway. [file 13071_2022_5427_MOESM3_ESM.docx]

**Table S3** **Antibodies targeting the key molecules in the host cell mitochondrial apoptosis signaling pathway**

| **Protein Name** | **Company** | **Host** | **Cat. No.** |
| --- | --- | --- | --- |
| BCL-2 | CST | M | 15071T |
| BCL-XL | ABCAM | M | ab77571 |
| BAD | ABCAM | R | ab32445 |
| BAX | ABCAM | M | ab3191 |
| Cytochrome C | ABCAM | R | ab133504 |
| MCL-1 | ABCAM | R | ab32087 |
| SURVIVIN  P53 | CST  CST | M  M | 2802T  2524T |

**CST:** Cell Signaling Technology; **R:** rabbit; **M:** mouse
